# (2,2′-Biquinoline-κ^2^ *N*,*N*′)dibromido­zinc(II)

**DOI:** 10.1107/S1600536810047434

**Published:** 2010-11-24

**Authors:** Hamideh Saravani, Ali Reza Rezvani, Niloufar Akbarzadeh Torbati

**Affiliations:** a Department of Chemistry, University of Sistan and Baluchestan, PO Box 98135-674, Zahedan, Iran

## Abstract

In the title compound, [ZnBr_2_(C_18_H_12_N_2_)], the Zn^II^ atom is four-coordinated in a distorted tetra­hedral configuration by two N atoms from the 2,2′-biquinoline ligand and two terminal Br atoms. The crystal packing is stabilized by weak inter­molecular C—H⋯Br hydrogen bonds and extensive inter­molecular π–π contacts between the pyridine and benzene rings [centroid–centroid distances = 3.775 (4), 3.748 (4), 3.735 (4), 3.538 (4), 3.678 (4) and 3.513 (4) Å].

## Related literature

For Zn—Br and Zn—N bond lengths in related structures, see: Alizadeh *et al.* (2009 ▶), Muranishi *et al.* (2005 ▶). For complexes of 2,2′-biquinoline, see: Bowmaker *et al.* (2005 ▶); Butcher & Sinn (1977 ▶); Kou *et al.* (2008 ▶); Moreno *et al.* (2007 ▶); Okabe & Muranishi (2005 ▶); Rahimi *et al.* (2009 ▶); Yoshikawa *et al.* (2003 ▶); Zhou & Ng (2006 ▶).
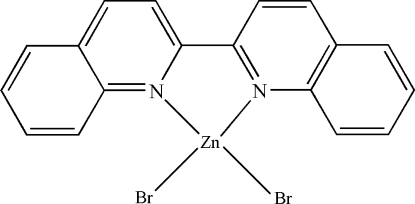


## Experimental

### 

#### Crystal data

[ZnBr_2_(C_18_H_12_N_2_)]
*M* *_r_* = 481.49
Monoclinic, 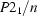

*a* = 7.9188 (16) Å
*b* = 12.351 (3) Å
*c* = 17.385 (4) Å
β = 103.01 (3)°
*V* = 1656.7 (7) Å^3^
*Z* = 4
Mo *K*α radiation
μ = 6.31 mm^−1^
*T* = 298 K
0.20 × 0.13 × 0.10 mm

#### Data collection

Bruker SMART CCD area-detector diffractometer
Absorption correction: multi-scan (*SADABS*; Sheldrick, 2000 ▶) *T*_min_ = 0.380, *T*_max_ = 0.530
13476 measured reflections
4471 independent reflections
2968 reflections with *I* > 2σ(*I*)
*R*_int_ = 0.098

#### Refinement

*R*[*F* ^2^ > 2σ(*F* ^2^)] = 0.070
*wR*(*F* ^2^) = 0.150
*S* = 1.15
4471 reflections
208 parameters
H-atom parameters constrained
Δρ_max_ = 1.14 e Å^−3^
Δρ_min_ = −0.69 e Å^−3^

### 

Data collection: *SMART* (Bruker, 1998 ▶); cell refinement: *SAINT* (Bruker, 1998 ▶); data reduction: *SAINT*; program(s) used to solve structure: *SHELXTL* (Sheldrick, 2008 ▶); program(s) used to refine structure: *SHELXTL*; molecular graphics: *ORTEP-3 for Windows* (Farrugia, 1997 ▶); software used to prepare material for publication: *WinGX* (Farrugia, 1999 ▶).

## Supplementary Material

Crystal structure: contains datablocks global, I. DOI: 10.1107/S1600536810047434/jj2062sup1.cif

Structure factors: contains datablocks I. DOI: 10.1107/S1600536810047434/jj2062Isup2.hkl

Additional supplementary materials:  crystallographic information; 3D view; checkCIF report

## Figures and Tables

**Table:** 

N1—Zn1	2.063 (4)
N2—Zn1	2.056 (5)
Zn1—Br2	2.3348 (11)
Zn1—Br1	2.3498 (12)

**Table:** 

N2—Zn1—N1	80.56 (18)
N2—Zn1—Br2	112.49 (14)
N1—Zn1—Br2	116.75 (13)
N2—Zn1—Br1	113.58 (14)
N1—Zn1—Br1	107.98 (15)
Br2—Zn1—Br1	119.24 (4)

**Table 2:** Hydrogen-bond geometry (Å, °)

*D*—H⋯*A*	*D*—H	H⋯*A*	*D*⋯*A*	*D*—H⋯*A*
C11—H11⋯Br2^i^	0.93	2.87	3.574 (7)	133

Symmetry code: (i) 
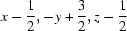
.

**Fig. 1.**
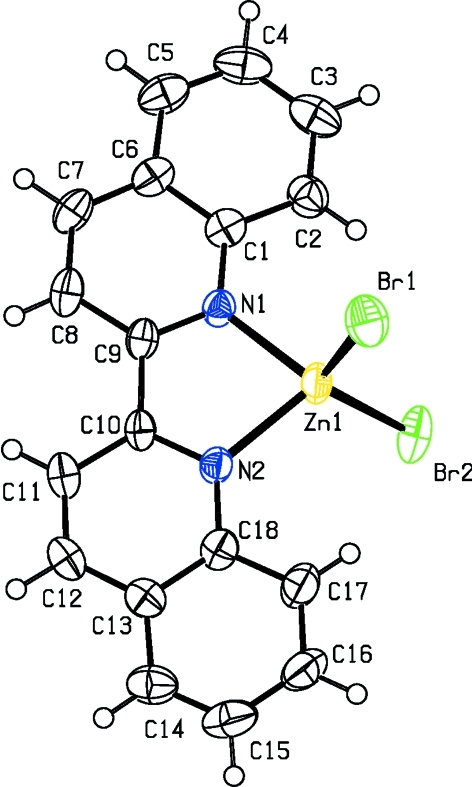
The molecular structure of the title molecule, with the atom-numbering scheme. Displacement ellipsoids are drawn at the 50% probability level.

**Fig. 2.**
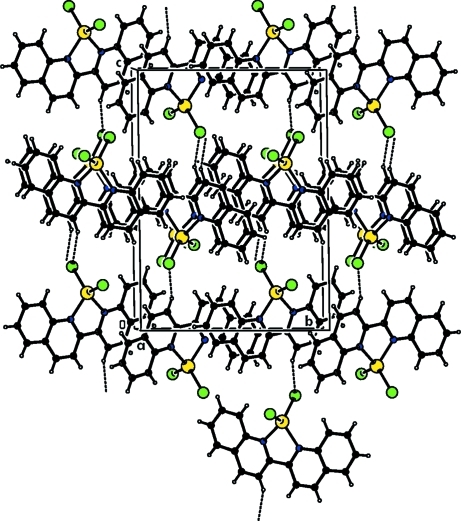
Packing diagram for title molecule viewed down the a axis. Dashed lines indicate weak C—H···Br intermolecular interactions.

## supplementary crystallographic information

### Comment

Numerous complexes have been prepared with the bidentate ligand 2,2'-biquinoline (2,2'-biq) such as that of iron (Rahimi *et al.*, 2009), iridium (Yoshikawa *et al.*, 2003), platinum (Okabe & Muranishi 2005), copper (Moreno *et al.*, 2007; Zhou & Ng 2006), silver (Bowmaker *et al.*, 2005), nickel (Kou *et al.*, 2008; Butcher & Sinn 1977) and palladium (Muranishi *et al.*, 2005). For further investigation of 2,2'-biquinoline, we have synthesized the title compound, [ZnBr_2_(C_18_H_12_N_2_)].

In the title compound, the Zn^II^ atom is four-coordinate in a distorted tetrahedral configuration with two N atoms from one 2,2'-biquinoline and two terminal Br atoms (Fig. 1).  The Zn—N and Zn—Br bond lengths and angles are within the normal ranges for [ZnCl_2_(biq)] (Muranishi *et al.*, 2005) and [ZnBr_2_(6,6'-dmbpy)], (Alizadeh *et al.*, 2009) [where 6,6'-dmbpy is 6,6'-dimethyl-2, 2'-bipyridine], respectively.  Crystal stability is enhanced by weak intermolecular C—H···Br hydrogen bonds (Table 2, Fig.2) and extensive weak π—π intermolecular contacts between the mean planes of the pyridine and phenyl rings (Table 3).

### Experimental

For the preparation of the title compound, a solution of 2,2'- biquinoline (0.51 g, 2.0 mmol) in methanol (10 ml) and chloroform (10 ml) was added to a solution of ZnBr_2_ (0.46 g, 2.0 mmol) in methanol (5 ml) and chloroform (5 ml) and the resulting solution was stirred for 20 min at room temperature. Suitable crystals for X-ray diffraction experiment were obtained by methanol diffusion into a solution in DMSO after one week (yield; 0.72 g, 74.8%).

### Refinement

All H atoms were positioned geometrically, with C—H = 0.93Å for aromatics (H) and constrained to ride on their parent atoms, with *U*_iso_(H) = 1.2*U*_eq_.

### Crystal data

**Table:**

[ZnBr_2_(C_18_H_12_N_2_)]	*F*(000) = 936
*M**_r_* = 481.49	*D*_x_ = 1.930 Mg m^−^^3^
Monoclinic, *P*2_1_/*n*	Mo *K*α radiation, λ = 0.71073 Å
Hall symbol: -P 2yn	Cell parameters from 434 reflections
*a* = 7.9188 (16) Å	θ = 2.0–29.3°
*b* = 12.351 (3) Å	µ = 6.31 mm^−^^1^
*c* = 17.385 (4) Å	*T* = 298 K
β = 103.01 (3)°	Block, colorless
*V* = 1656.7 (7) Å^3^	0.20 × 0.13 × 0.10 mm
*Z* = 4	

### Data collection

**Table:**

Bruker SMART CCD area-detector diffractometer	4471 independent reflections
Radiation source: fine-focus sealed tube	2968 reflections with *I* > 2σ(*I*)
graphite	*R*_int_ = 0.098
θ and ω scans	θ_max_ = 29.3°, θ_min_ = 2.0°
Absorption correction: multi-scan (*SADABS*; Sheldrick, 2000)	*h* = −9→10
*T*_min_ = 0.380, *T*_max_ = 0.530	*k* = −16→16
13476 measured reflections	*l* = −23→23

### Refinement

**Table:**

Refinement on *F*^2^	Primary atom site location: structure-invariant direct methods
Least-squares matrix: full	Secondary atom site location: difference Fourier map
*R*[*F*^2^ > 2σ(*F*^2^)] = 0.070	Hydrogen site location: inferred from neighbouring sites
*wR*(*F*^2^) = 0.150	H-atom parameters constrained
*S* = 1.15	*w* = 1/[σ^2^(*F*_o_^2^) + (0.0538*P*)^2^ + 1.2857*P*] where *P* = (*F*_o_^2^ + 2*F*_c_^2^)/3
4471 reflections	(Δ/σ)_max_ = 0.007
208 parameters	Δρ_max_ = 1.14 e Å^−^^3^
0 restraints	Δρ_min_ = −0.69 e Å^−^^3^

### Special details

**Table:**

Geometry. All e.s.d.'s (except the e.s.d. in the dihedral angle between two l.s. planes) are estimated using the full covariance matrix. The cell e.s.d.'s are taken into account individually in the estimation of e.s.d.'s in distances, angles and torsion angles; correlations between e.s.d.'s in cell parameters are only used when they are defined by crystal symmetry. An approximate (isotropic) treatment of cell e.s.d.'s is used for estimating e.s.d.'s involving l.s. planes.
Refinement. Refinement of *F*^2^ against ALL reflections. The weighted *R*-factor *wR* and goodness of fit *S* are based on *F*^2^, conventional *R*-factors *R* are based on *F*, with *F* set to zero for negative *F*^2^. The threshold expression of *F*^2^ > σ(*F*^2^) is used only for calculating *R*-factors(gt) *etc*. and is not relevant to the choice of reflections for refinement. *R*-factors based on *F*^2^ are statistically about twice as large as those based on *F*, and *R*- factors based on ALL data will be even larger.

### Fractional atomic coordinates and isotropic or equivalent isotropic displacement parameters (Å^2^)

**Table:**

	*x*	*y*	*z*	*U*_iso_*/*U*_eq_	
C1	0.7304 (8)	0.5440 (4)	0.0324 (4)	0.0354 (13)	
C2	0.8156 (9)	0.4877 (5)	0.1012 (4)	0.0434 (14)	
H2	0.8158	0.5152	0.1510	0.052*	
C3	0.8975 (9)	0.3922 (5)	0.0933 (5)	0.0518 (18)	
H3	0.9539	0.3547	0.1382	0.062*	
C4	0.8974 (10)	0.3501 (5)	0.0183 (5)	0.0529 (18)	
H4	0.9541	0.2851	0.0142	0.063*	
C5	0.8161 (10)	0.4024 (5)	−0.0483 (5)	0.0488 (17)	
H5	0.8161	0.3728	−0.0975	0.059*	
C6	0.7312 (8)	0.5020 (5)	−0.0430 (4)	0.0386 (13)	
C7	0.6441 (9)	0.5609 (5)	−0.1093 (4)	0.0428 (15)	
H7	0.6430	0.5354	−0.1598	0.051*	
C8	0.5609 (9)	0.6557 (5)	−0.1000 (3)	0.0424 (15)	
H8	0.5021	0.6947	−0.1436	0.051*	
C9	0.5666 (8)	0.6924 (5)	−0.0231 (3)	0.0317 (11)	
C10	0.4705 (8)	0.7925 (4)	−0.0082 (3)	0.0313 (12)	
C11	0.3551 (8)	0.8467 (5)	−0.0691 (4)	0.0383 (13)	
H11	0.3384	0.8227	−0.1210	0.046*	
C12	0.2681 (8)	0.9347 (5)	−0.0514 (4)	0.0409 (14)	
H12	0.1921	0.9716	−0.0915	0.049*	
C13	0.2924 (8)	0.9702 (5)	0.0272 (4)	0.0387 (13)	
C14	0.2045 (10)	1.0612 (5)	0.0494 (5)	0.0489 (17)	
H14	0.1258	1.0995	0.0113	0.059*	
C15	0.2351 (11)	1.0923 (6)	0.1259 (5)	0.058 (2)	
H15	0.1752	1.1510	0.1401	0.070*	
C16	0.3560 (12)	1.0371 (6)	0.1841 (5)	0.0576 (19)	
H16	0.3777	1.0610	0.2361	0.069*	
C17	0.4432 (10)	0.9479 (6)	0.1651 (4)	0.0495 (17)	
H17	0.5223	0.9110	0.2039	0.059*	
C18	0.4100 (8)	0.9135 (5)	0.0853 (3)	0.0369 (13)	
N1	0.6500 (7)	0.6402 (4)	0.0408 (3)	0.0312 (10)	
N2	0.4991 (7)	0.8251 (4)	0.0668 (3)	0.0320 (10)	
Zn1	0.67435 (10)	0.73002 (6)	0.14281 (4)	0.03680 (19)	
Br1	0.95564 (10)	0.80280 (6)	0.17474 (5)	0.0561 (2)	
Br2	0.55785 (11)	0.65496 (6)	0.24275 (4)	0.0554 (2)	

### Atomic displacement parameters (Å^2^)

**Table:**

	*U*^11^	*U*^22^	*U*^33^	*U*^12^	*U*^13^	*U*^23^
C1	0.039 (3)	0.029 (3)	0.041 (3)	−0.006 (2)	0.014 (3)	0.000 (2)
C2	0.043 (4)	0.044 (3)	0.044 (3)	0.001 (3)	0.010 (3)	0.007 (3)
C3	0.042 (4)	0.039 (3)	0.074 (5)	0.005 (3)	0.011 (4)	0.013 (3)
C4	0.045 (4)	0.031 (3)	0.086 (6)	0.001 (3)	0.019 (4)	−0.007 (3)
C5	0.053 (4)	0.039 (3)	0.059 (4)	−0.008 (3)	0.022 (4)	−0.016 (3)
C6	0.039 (3)	0.037 (3)	0.042 (3)	−0.008 (3)	0.015 (3)	−0.008 (3)
C7	0.055 (4)	0.039 (3)	0.035 (3)	−0.011 (3)	0.011 (3)	−0.016 (3)
C8	0.051 (4)	0.046 (3)	0.027 (3)	−0.011 (3)	0.003 (3)	−0.003 (3)
C9	0.035 (3)	0.032 (3)	0.026 (2)	−0.010 (2)	0.003 (2)	−0.003 (2)
C10	0.035 (3)	0.031 (3)	0.025 (2)	−0.006 (2)	0.001 (2)	0.001 (2)
C11	0.040 (3)	0.040 (3)	0.030 (3)	−0.007 (3)	−0.002 (2)	0.005 (2)
C12	0.037 (3)	0.039 (3)	0.042 (3)	0.001 (3)	0.000 (3)	0.010 (3)
C13	0.034 (3)	0.037 (3)	0.044 (3)	−0.002 (2)	0.008 (3)	0.003 (3)
C14	0.050 (4)	0.035 (3)	0.066 (5)	0.004 (3)	0.022 (4)	0.004 (3)
C15	0.063 (5)	0.052 (4)	0.071 (5)	0.009 (4)	0.039 (4)	−0.001 (4)
C16	0.074 (5)	0.058 (4)	0.046 (4)	0.010 (4)	0.025 (4)	−0.011 (3)
C17	0.065 (5)	0.052 (4)	0.034 (3)	0.003 (3)	0.015 (3)	−0.002 (3)
C18	0.046 (4)	0.030 (3)	0.035 (3)	−0.005 (2)	0.011 (3)	0.001 (2)
N1	0.038 (3)	0.028 (2)	0.027 (2)	−0.0014 (19)	0.0052 (19)	−0.0017 (18)
N2	0.040 (3)	0.030 (2)	0.026 (2)	0.002 (2)	0.0074 (19)	0.0012 (18)
Zn1	0.0467 (4)	0.0377 (3)	0.0234 (3)	0.0022 (3)	0.0023 (3)	0.0002 (3)
Br1	0.0502 (4)	0.0611 (4)	0.0508 (4)	−0.0085 (3)	−0.0019 (3)	−0.0033 (3)
Br2	0.0760 (5)	0.0629 (4)	0.0263 (3)	−0.0154 (4)	0.0095 (3)	0.0018 (3)

### Geometric parameters (Å, °)

**Table:**

C1—N1	1.371 (7)	C11—C12	1.359 (9)
C1—C6	1.412 (8)	C11—H11	0.9300
C1—C2	1.416 (9)	C12—C13	1.407 (9)
C2—C3	1.368 (10)	C12—H12	0.9300
C2—H2	0.9300	C13—C18	1.399 (9)
C3—C4	1.404 (11)	C13—C14	1.420 (9)
C3—H3	0.9300	C14—C15	1.353 (11)
C4—C5	1.355 (11)	C14—H14	0.9300
C4—H4	0.9300	C15—C16	1.403 (12)
C5—C6	1.415 (9)	C15—H15	0.9300
C5—H5	0.9300	C16—C17	1.379 (10)
C6—C7	1.405 (9)	C16—H16	0.9300
C7—C8	1.371 (10)	C17—C18	1.417 (9)
C7—H7	0.9300	C17—H17	0.9300
C8—C9	1.403 (8)	C18—N2	1.376 (8)
C8—H8	0.9300	N1—Zn1	2.063 (4)
C9—N1	1.325 (7)	N2—Zn1	2.056 (5)
C9—C10	1.504 (8)	Zn1—Br2	2.3348 (11)
C10—N2	1.334 (7)	Zn1—Br1	2.3498 (12)
C10—C11	1.404 (8)		
			
N1—C1—C6	121.1 (6)	C11—C12—C13	120.2 (6)
N1—C1—C2	118.8 (6)	C11—C12—H12	119.9
C6—C1—C2	120.1 (6)	C13—C12—H12	119.9
C3—C2—C1	119.1 (7)	C18—C13—C12	118.0 (6)
C3—C2—H2	120.4	C18—C13—C14	119.2 (6)
C1—C2—H2	120.4	C12—C13—C14	122.9 (6)
C2—C3—C4	120.7 (7)	C15—C14—C13	120.1 (7)
C2—C3—H3	119.6	C15—C14—H14	119.9
C4—C3—H3	119.6	C13—C14—H14	119.9
C5—C4—C3	121.2 (6)	C14—C15—C16	120.8 (7)
C5—C4—H4	119.4	C14—C15—H15	119.6
C3—C4—H4	119.4	C16—C15—H15	119.6
C4—C5—C6	120.1 (7)	C17—C16—C15	120.9 (7)
C4—C5—H5	120.0	C17—C16—H16	119.6
C6—C5—H5	120.0	C15—C16—H16	119.6
C7—C6—C1	117.9 (5)	C16—C17—C18	118.8 (7)
C7—C6—C5	123.3 (6)	C16—C17—H17	120.6
C1—C6—C5	118.8 (6)	C18—C17—H17	120.6
C8—C7—C6	120.3 (6)	N2—C18—C13	121.4 (5)
C8—C7—H7	119.8	N2—C18—C17	118.3 (6)
C6—C7—H7	119.8	C13—C18—C17	120.2 (6)
C7—C8—C9	118.4 (6)	C9—N1—C1	119.3 (5)
C7—C8—H8	120.8	C9—N1—Zn1	113.0 (4)
C9—C8—H8	120.8	C1—N1—Zn1	127.0 (4)
N1—C9—C8	123.0 (6)	C10—N2—C18	119.0 (5)
N1—C9—C10	115.6 (5)	C10—N2—Zn1	113.4 (4)
C8—C9—C10	121.4 (5)	C18—N2—Zn1	127.6 (4)
N2—C10—C11	122.1 (5)	N2—Zn1—N1	80.56 (18)
N2—C10—C9	115.8 (5)	N2—Zn1—Br2	112.49 (14)
C11—C10—C9	122.0 (5)	N1—Zn1—Br2	116.75 (13)
C12—C11—C10	119.2 (6)	N2—Zn1—Br1	113.58 (14)
C12—C11—H11	120.4	N1—Zn1—Br1	107.98 (15)
C10—C11—H11	120.4	Br2—Zn1—Br1	119.24 (4)
			
N1—C1—C2—C3	−179.4 (6)	C12—C13—C18—C17	178.1 (6)
C6—C1—C2—C3	−0.3 (9)	C14—C13—C18—C17	−1.2 (9)
C1—C2—C3—C4	−0.1 (10)	C16—C17—C18—N2	179.3 (6)
C2—C3—C4—C5	−0.2 (11)	C16—C17—C18—C13	0.8 (10)
C3—C4—C5—C6	0.8 (11)	C8—C9—N1—C1	−2.2 (9)
N1—C1—C6—C7	−1.2 (9)	C10—C9—N1—C1	175.3 (5)
C2—C1—C6—C7	179.8 (6)	C8—C9—N1—Zn1	168.5 (5)
N1—C1—C6—C5	180.0 (6)	C10—C9—N1—Zn1	−13.9 (6)
C2—C1—C6—C5	0.9 (9)	C6—C1—N1—C9	2.4 (8)
C4—C5—C6—C7	−179.9 (7)	C2—C1—N1—C9	−178.5 (6)
C4—C5—C6—C1	−1.2 (10)	C6—C1—N1—Zn1	−166.9 (4)
C1—C6—C7—C8	−0.4 (9)	C2—C1—N1—Zn1	12.2 (8)
C5—C6—C7—C8	178.4 (6)	C11—C10—N2—C18	1.3 (8)
C6—C7—C8—C9	0.6 (9)	C9—C10—N2—C18	−177.3 (5)
C7—C8—C9—N1	0.7 (9)	C11—C10—N2—Zn1	180.0 (4)
C7—C8—C9—C10	−176.7 (6)	C9—C10—N2—Zn1	1.4 (6)
N1—C9—C10—N2	8.6 (7)	C13—C18—N2—C10	−0.8 (8)
C8—C9—C10—N2	−173.8 (5)	C17—C18—N2—C10	−179.3 (6)
N1—C9—C10—C11	−170.0 (5)	C13—C18—N2—Zn1	−179.3 (4)
C8—C9—C10—C11	7.6 (9)	C17—C18—N2—Zn1	2.3 (8)
N2—C10—C11—C12	−0.6 (9)	C10—N2—Zn1—N1	−6.6 (4)
C9—C10—C11—C12	177.9 (5)	C18—N2—Zn1—N1	171.8 (5)
C10—C11—C12—C13	−0.6 (9)	C10—N2—Zn1—Br2	−121.8 (4)
C11—C12—C13—C18	1.1 (9)	C18—N2—Zn1—Br2	56.7 (5)
C11—C12—C13—C14	−179.6 (6)	C10—N2—Zn1—Br1	99.0 (4)
C18—C13—C14—C15	0.2 (10)	C18—N2—Zn1—Br1	−82.6 (5)
C12—C13—C14—C15	−179.1 (7)	C9—N1—Zn1—N2	11.4 (4)
C13—C14—C15—C16	1.3 (12)	C1—N1—Zn1—N2	−178.7 (5)
C14—C15—C16—C17	−1.8 (13)	C9—N1—Zn1—Br2	121.9 (4)
C15—C16—C17—C18	0.7 (12)	C1—N1—Zn1—Br2	−68.2 (5)
C12—C13—C18—N2	−0.4 (9)	C9—N1—Zn1—Br1	−100.4 (4)
C14—C13—C18—N2	−179.7 (5)	C1—N1—Zn1—Br1	69.4 (5)

### Hydrogen-bond geometry (Å, °)

**Table:**

*D*—H···*A*	*D*—H	H···*A*	*D*···*A*	*D*—H···*A*
C11—H11···Br2^i^	0.93	2.87	3.574 (7)	133

Symmetry codes: (i) *x*−1/2, −*y*+3/2, *z*−1/2.

### Table 3 Cg···Cg π—π interactions [Cg1, Cg2, Cg3, Cg5, Cg5 = centroids of rings Zn1/N1/C9/C10/N2; N1/C1/C6/C7/C8/C9; N2/C10/C11/C12/C13/C18; C1–C6; C13–C18; Symmetry codes: (i) 1-x, 1-y, -z; (ii) 1-x, 2-y, -z; (iii) 2-x, 1-y, -z] Cg(I) Cg(J) Cg···Cg (Å)

**Table:**

Cg1	Cg4	3.775 (4)^i^
Cg2	Cg2	3.748 (4)^i^
Cg2	Cg4	3.735 (4)^i^
Cg3	Cg3	3.538 (4)^ii^
Cg3	Cg5	3.678 (4)^ii^
Cg4	Cg4	3.513 (4)^iii^
